# FERMT1 knockdown inhibits oral squamous cell carcinoma cell epithelial-mesenchymal transition by inactivating the PI3K/AKT signaling pathway

**DOI:** 10.1186/s12903-021-01955-9

**Published:** 2021-11-23

**Authors:** Xiao Wang, Qianqian Chen

**Affiliations:** 1 Department of Stomatology, The First Affiliated Hospital, Guangxi University of Science and Technology, Liuzhou, 545006 China; 2 Medical College, Medical Experimental Center, Guangxi University of Science and Technology, Building D, 257 Liushi Road, Yufeng District, Liuzhou, 545006 China

**Keywords:** FERMT1, Invasion, Migration, Oral squamous cell cancer

## Abstract

**Background:**

The metastasis of oral cancer is one of the main causes of death. However, the mechanisms underlying oral cancer metastasis have not been completely elucidated. Fermitin family member 1 (FERMT1) plays an -oncogene role in many cancers; however, the role of FERMT1 in oral squamous cell cancer (OSCC) remains unclear.

**Methods:**

In this study, OSCC cells were treated with 5 ng/ml recombinant human Transforming growth factor-β1 (TGF-β1) protein. FERMT1 expression was measured in OSCC cell lines by RT-qPCR and western blotting. The effect of FERMT1 knockdown on the migration and invasion of OSCC cells was evaluated by Transwell assay. The epithelial-mesenchymal transition (EMT) and PI3K/AKT signaling pathway-related mRNA expression and protein levels were assessed by RT-qPCR and western blotting.

**Results:**

We found that FERMT1 expression was elevated in TGF-β1-induced OSCC cell lines, and knockdown of FERMT1 inhibited the migration and invasion in TGF-β1-induced OSCC cells. FERMT1 silencing inhibited vimentin, N-cadherin, matrix metalloproteinase 9 (MMP-9) expression and promoted E-cadherin expression, suggesting that FERMT1 silencing inhibited EMT in TGF-β1-induced OSCC cells. Furthermore, FERMT1 silencing inactivated the PI3K/AKT signaling pathway in TGF-β1-induced OSCC cells. Activation of the PI3K/AKT signaling pathway reversed the effect of FERMT1 silencing on OSCC cell migration, invasion, and EMT.

**Conclusions:**

FERMT1 silencing inhibits the migration, invasion, and EMT of OSCC cells via inactivation of the PI3K/AKT signaling pathway, suggesting that FERMT1 is a novel and potential therapeutic target for anti-metastatic strategies for OSCC.

## Background

Oral cancer is the eighth most common type of cancer worldwide, and approximately 90% of all oral malignancies are oral squamous cell cancer (OSCC) [1]. In China, the overall crude incidence rate for oral cancer was 2.93/100,000 in 2011 [2]. Smoking, alcohol drinking, human papilloma virus, serum excess and deficient levels of Cu or Zn, and betel quid (BQ) chewing play key roles in the development of oral cancer [3–7]. At present, the mortality rate of oral cancer remains high, and the primary cause is tumor metastasis [8, 9]. Hence, knowing what genes can inhibit oral cancer metastasis and EMT can help the development of therapeutic targets.

Epithelial-mesenchymal transition (EMT) occurrence help the OSCC metastasis [10, 11]. During EMT, epithelial cells lose their junctions and increase their motility, which promotes epithelial cells to leave the tissue and enter the systemic circulation, thereby enhancing the development of cancer metastasis [12]. During EMT, there is a decrease in the expression of epithelial markers, including E-cadherin, tight junction proteins, and cytokeratin, as well as increase in the expression of mesenchymal markers, including vimentin, fibronectin, α-smooth muscle actin, N-cadherin [12]. EMT programs can be activate by activated PI3K/Akt pathway in oral cancer [13, 14]. PI3K/Akt pathway play a key role in regulating cell growth and metabolism in normal physiology. PI3K can activate by insulin-like growth factor 1 receptor, then induced phosphorylation of AKT [15–17]. AKT which is a central mediator of the PI3K pathway can further activate the phosphorylation of mTOR and NF-κB and regulate eukaryotic translation initiation factor 4E and ribosomal protein S6 kinase in cancer [15–17]. Hence, excessively increased PI3K expression and the level of phosphorylated AKT/total AKT (p-AKT/AKT) indicates activation of the PI3K/AKT signaling pathway. In addition, the activation of EMT programs involves many proteins, such as fermitin family member 1 (FERMT1). FERMT1, which encodes the kindlin-1 protein that belongs to a family of focal adhesion proteins, activates EMT to promote colon cancer metastasis both in vitro and in vivo [18]. However, whether FERMT1 can promote OSCC metastasis and EMT by activating PI3K/AKT have not been explored.

In OSCC, EMT was induced by TGF-β1 [19–21]. In this study, FERMT1 expression was measured in OSCC cell lines with or without TGF-β1 induction. Additionally, the effect of FERMT1 knockdown on the migration, invasion, EMT, and PI3K/Akt pathway in OSCC cells were studied. Our research explored and confirmed the role of FERMT1 as a therapeutic target for OSCC metastasis.

## Materials and methods

### Cell culture

Human oral keratinocytes (HOK) and OSCC cell lines include CAL-27, Tca8113, and SCC15 (China Center for Type Culture Collection, Wuhan, China) were cultured in the special keratinocyte growth medium (Clonetics, San Diego, CA, USA) and the Dulbecco’s modified Eagle medium supplemented with 10% fetal bovine serum, respectively. All cells were treated with 5 ng/ml recombinant human TGF-β1 protein (T & L biological technology, Beijing, China).

### Reverse transcription-quantitative polymerase chain reaction (RT-qPCR)

Total RNA was extracted from TGF-β1-treated Tca8113 and SCC15 cells using the TRIzol® reagent (Invitrogen; Thermo Fisher Scientific, Inc., Foster City, CA, USA), reversed transcription using the PrimeScript RT Reagent kit, and FERMT1 expression was measured by RT-qPCR using SYBR Premix Ex Taq reagent (Takara, Dalian, China). RT-qPCR was performed using an Applied Biosystems 7500 system (ThermoFisher Scientific). The primer sequences are presented in Table 1. Gene expression levels were quantified using the 2^−ΔΔCt^ method, and normalized to the expression of the control, GAPDH (13).

**Table 1:** The primer sequences

Gene	Forward primer (5' -3')	Reverse primer (5' -3')	Size
FERMT1	TAAACTTGCAGATAATCTCA	CAAGTTCCTTATTTTTAAAG	118 bp
PI3K(PIK3CB)	TAATCGGAGGATAGGGCAGT	TTCATGTGCCCCACACTTCC	123 bp
AKT (AKT1)	GGTGATCCTGGTGAAGGAGA	AAGGGGTGCCTGGAGTTCTG	138 bp
MMP-9	TCTGCCTGCACCACCGACG	CTGGGTGTAGAGTCTCTCG	114 bp
E-cadherin	CAACGATAATCCTCCGATCT	ACGGTGACGGTGGCTGTGGA	138 bp
vimentin	GAAGAGGAAATCCAGGAGCT	TTTCATATTGCTGACGTACGT	118 bp
N-cadherin	TAATGGAAATCAAGT	ATCCCTCAGGAACTGTCCCA	113 bp
GAPDH	GCTCATTTGCAGGGGGGAG	GTTGGTGGTGCAGGAGGCA	138 bp

### Western blotting

FERMT1, PI3K, AKT, p-AKT, MMP-9, E-cadherin, vimentin, and N-cadherin expression were measured by western blotting according to previous study [21]. Briefly, the total protein was extracted from TGF-β1-treated Tca8113 and SCC15 cells using radioimmunoprecipitation assay buffer (Takara), separated by 8% SDS-PAGE, and then transferred onto polyvinylidene difluoride membranes. After blocking, the membranes were incubated with rabbit anti-human FERMT1 (ab68041), PI3K (ab140307), AKT (ab8805), p-AKT (ab38449), MMP-9 (ab76003), E-cadherin (ab76055), vimentin (ab92547), N-cadherin (ab76011), and GAPDH (ab8245) antibody (Abcam, Cambridge, MA, USA) for 1 h at 37 °C. After wash, membranes were incubated with goat anti-rabbit secondary antibody (a horseradish peroxidase-conjugated IgG H&L, Abcam) for 40 min, and proteins were visualized using an enhanced chemiluminescence reagent (Thermo Fisher Scientific, Inc.). The expression levels of the proteins of interest were normalized (Image J 6.0, National Institutes of Health, Bethesda, MD, USA) against the expression levels of β-actin.

### Transfection with si-FERMT1 and IGF-1 treatment

siRNA negative control (si-NC, 5′-UUCUCCGAACGUGUCACGUTT-3′) and si-FERMT1 (si-FERMT1-1: 5′-GAAACAAGUGCUAAGUGUACC-3′; si-FERMT1-2: 5′-CUAUUUCUCAGUUCUAUUAUU-3′; si-FERMT1-3: 5′-GGGAAAUAUCAGACAAUAUUU-3′) were purchased from GenePharma (Suzhou, China). Cells were transfected with 20, 50, and 100 nM siRNAs using the Lipofectamine® 2000 reagent (Invitrogen; Thermo Fisher Scientific). Then the si-FERMT1-transfected cells were treated with PBS or insulin-like growth factor-1 (IGF-1).

### Transwell migration and invasion assays

Cell migration and invasion were assessed using the Transwell assay with or without Matrigel pre-coating (BD Biosciences), respectively, according to previous study [21]. After culturing for 24 h, cells from the bottom chamber were stained with 0.1% crystal violet solution in 20% ethanol, and counted using a phase contrast light microscope (Olympus Corporation, Tokyo, Japan). The migrated and invasive cells were calculated in 5 randomly selected high-power fields and the mean was as the final result.

### Statistical analysis

Data in this study conformed to normal distribution are presented as mean ± standard deviation. The differences between groups were performed using the SPSS software version 19.0 (IBM SPSS, Armonk, NY, USA). The differences between the two groups were used to analyze an independent t-test. While the differences between three or more groups were analyzed using a one-way analysis of variance, followed by a post-hoc LSD test. *P* < 0.05 has a significant difference.

## Results

### FERMT1 expression is promoted in in TGF-β1-induced OSCC cell lines

**Fig. 1:**
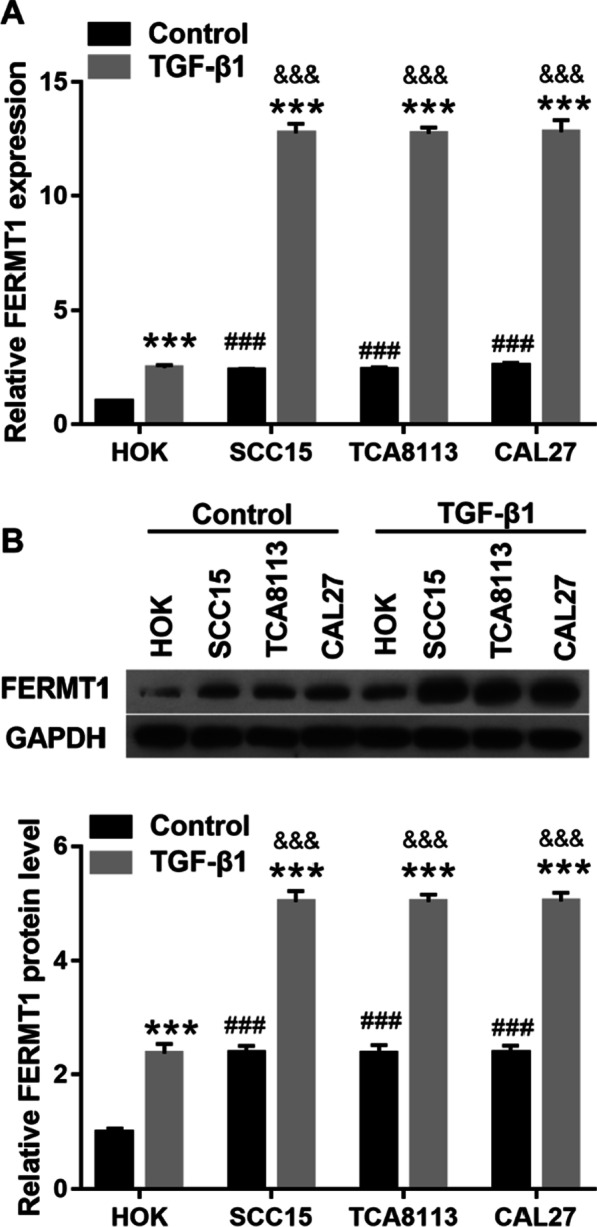
FERMT1 expression is promoted in oral squamous cell cancer cell lines. OSCC cells and HOK cell were treated with 5 ng/ml recombinant human TGF-β1 protein. FERMT1 expression was analyzed by RT-qPCR (**a**) and western blotting (**b**). ***P < 0.001 TGF-β1 treatment group vs control group. ^###^*P* < 0.001, OSCC cells vs HOK cell in control group. ^&&&^*P* < 0.001, OSCC cells vs HOK cell in TGF-β1 treatment group. HOK: human oral keratinocytes; CAL-27, Tca8113, and SCC15: oral squamous cell cancer cell lines

FERMT1 expression was measured by RT-qPCR and western blotting (Fig. 1). Compared with control group, FERMT1 mRNA expression and protein level was significantly enhanced in the in TGF-β1-induced HOK, CAL-27, Tca8113, and SCC15. Compared with HOK in control group, FERMT1 expression was significantly enhanced in the in OSCC cell lines CAL-27, Tca8113, and SCC15 in control group. In addition, FERMT1 expression was significantly enhanced in the in TGF-β1-induced OSCC cell lines CAL-27, Tca8113, and SCC15 compared with TGF-β1-induced HOK. FERMT1 expression did not differ significantly between the three OSCC cell lines. Hence, Tca8113 and SCC15 cells were selected for further experiments.

### Knockdown of FERMT1 suppresses migration and invasion in TGF-β1-induced OSSC cells

**Fig. 2:**
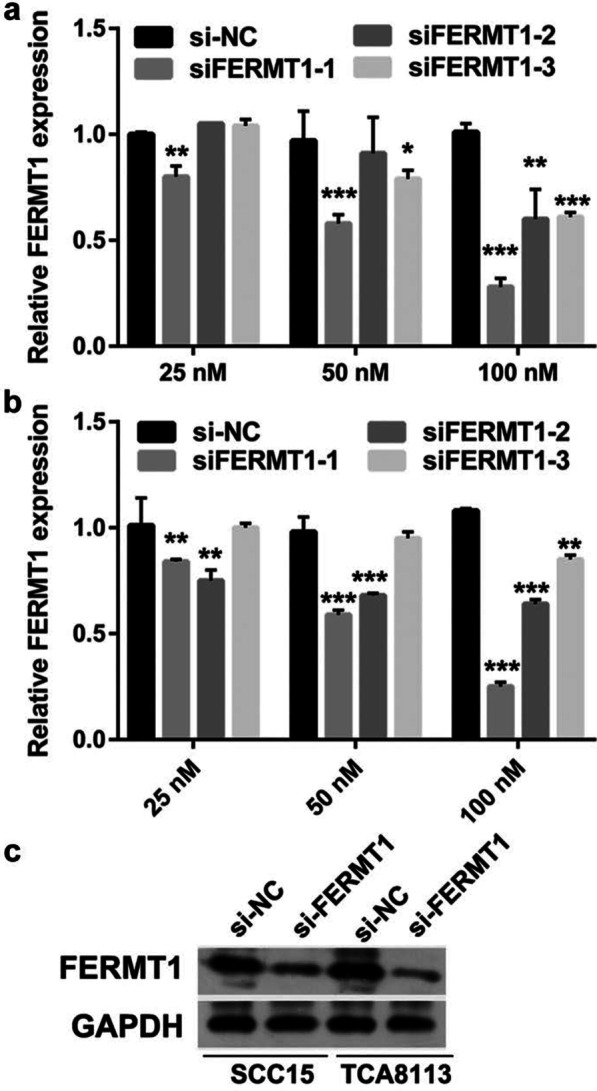
FERMT1 expression was silenced by si-FERMT1 transfection. OSCC cells were treated with 5 ng/ml recombinant human TGF-β1 protein. (**a** and **b**) FERMT1 expression was analyzed by RT-qPCR after transfection of three si-FERMT1s at 24 h in SCC15 (**a**) and Tca8113 (**b**) cells. (**c**) FERMT1 expression was analyzed by western blotting. *P < 0.05, **P < 0.01, and ***P < 0.001 vs. si-NC

**Fig. 3:**
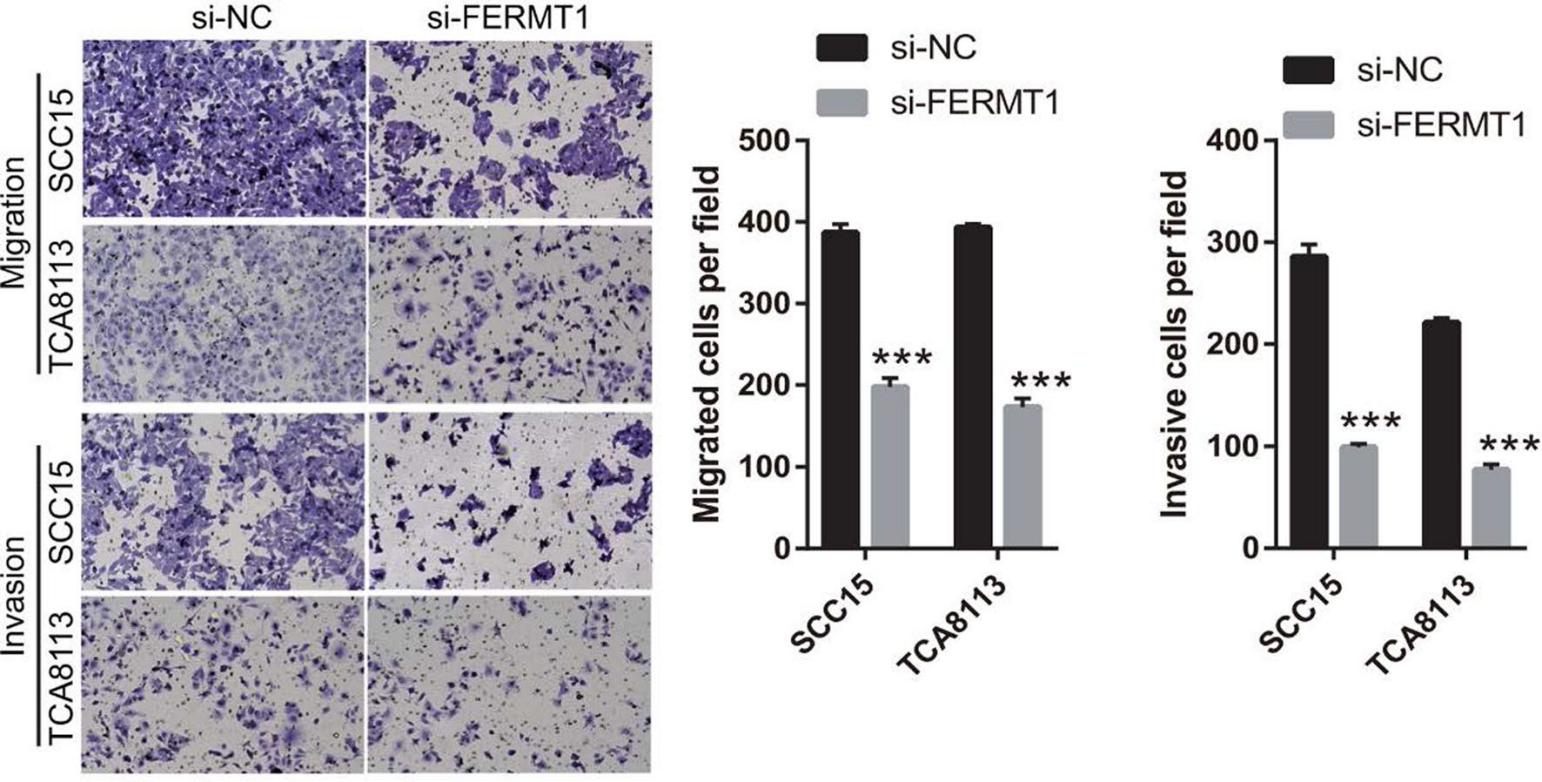
Knockdown of FERMT1 suppresses migration and invasion of OSSC cells. OSCC cells were treated with 5 ng/ml recombinant human TGF-β1 protein. The migration and invasion of OSSC cells were measured by Transwell (× 200). ***P < 0.001

**Fig. 4:**
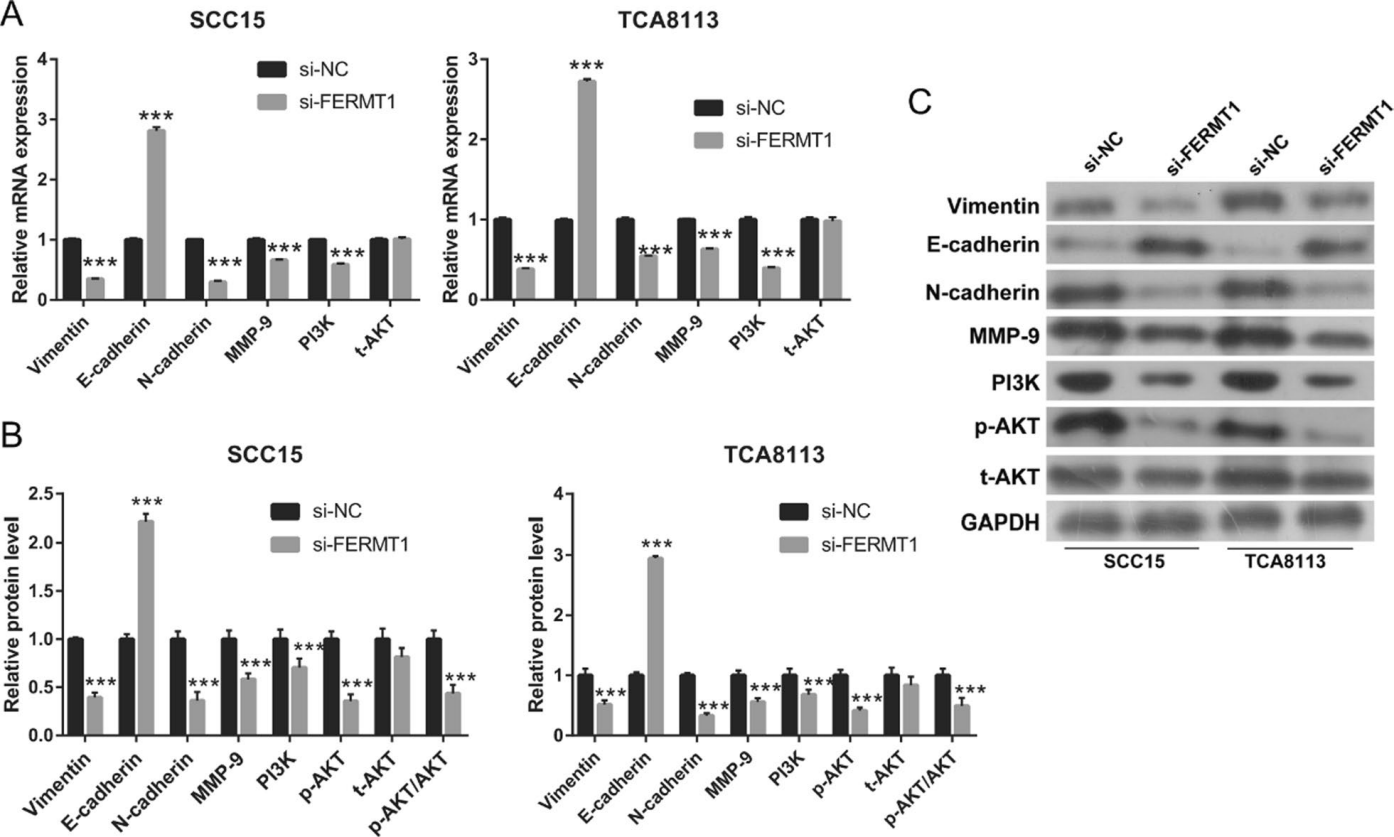
Knockdown FERMT1 inhibited EMT and the PI3K/AKT signaling pathway. OSCC cells were treated with 5 ng/ml recombinant human TGF-β1 protein. (**A**, **B**) mRNA Expression (**A**) and protein levels (**B**) of mesenchymal biomarkers including vimentin, N-cadherin, and MMP-9, epithelial biomarkers including E-cadherin, and PI3K, total (t)-AKT, and phosphorylated (p)-AKT were measured by qRT-PCR and western blotting after transfection in Tca8113 and SCC15 cells at 24 h. (**C**) The representative image of western blot

To assess the effect of FERMT1 on OSCC cell migration and invasion, si-FERMT1s were transfected into Tca8113 and SCC15 cells, and FERMT1 expression was measured by RT-qPCR and western blotting (Fig. 2). Compared with other groups, FERMT1 expression was significantly inhibited in the 100-nM si-FERMT1-1 (named si-FERMT1 in further experiments) group. Following transfection of si-FERMT1 or si-NC at 48 h, the migration and invasion of Tca8113 and SCC15 cells was significantly reduced in the si-FERMT1 group compared with the si-NC group (Fig. 3). Additionally, western blotting results showed that si-FERMT1 transfection evidently inhibited vimentin, N-cadherin, and MMP-9 mRNA expression and protein level while promoted E-cadherin mRNA expression and protein level compared with si-NC-transfected Tca8113 and SCC15 cells (Fig. 4).

### Knockdown of FERMT1 silences the PI3K/AKT signaling pathway

After transfection with si-FERMT1 and si-NC, the PI3K mRNA expression was markedly lower while t-AKT had no marked change in si-FERMT1-transfected Tca8113 and SCC15 cells compared with that in si-NC transfected Tca8113 and SCC15 cells. In addition, the protein levels of PI3K and p-AKT and p-AKT/t-AKT were markedly lower while t-AKT had no marked change in si-FERMT1-transfected Tca8113 and SCC15 cells compared with those in si-NC transfected Tca8113 and SCC15 cells (Fig. 4).

### Activated PI3K/AKT signaling pathway reverses the effect of FERMT1 on OSSC cell migration and invasion

**Fig. 5:**
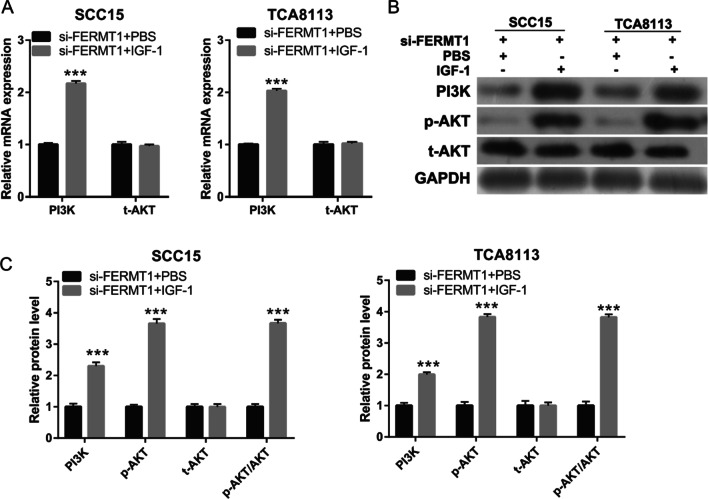
IGF-1 treatment activated the PI3K/AKT signaling pathway in si-FERMT1-transfected Tca8113 and SCC15 cells. OSCC cells were treated with 5 ng/ml recombinant human TGF-β1 protein. **A** PI3K, total (t)-AKT mRNA expression were analyzed by qRT-PCR after IGF-1 treatment at 24 h in si-FERMT1-transfected Tca8113 and SCC15 cells. **B** The representative image of western blot. **C** PI3K, t-AKT, and phosphorylated (p)-AKT protein levels were analyzed by western blotting after IGF-1 treatment at 24 h in si-FERMT1-transfected Tca8113 and SCC15 cells. ***P < 0.001, si-FERMT1 + IGF-1 vs si-FERMT1 + PBS

**Fig. 6:**
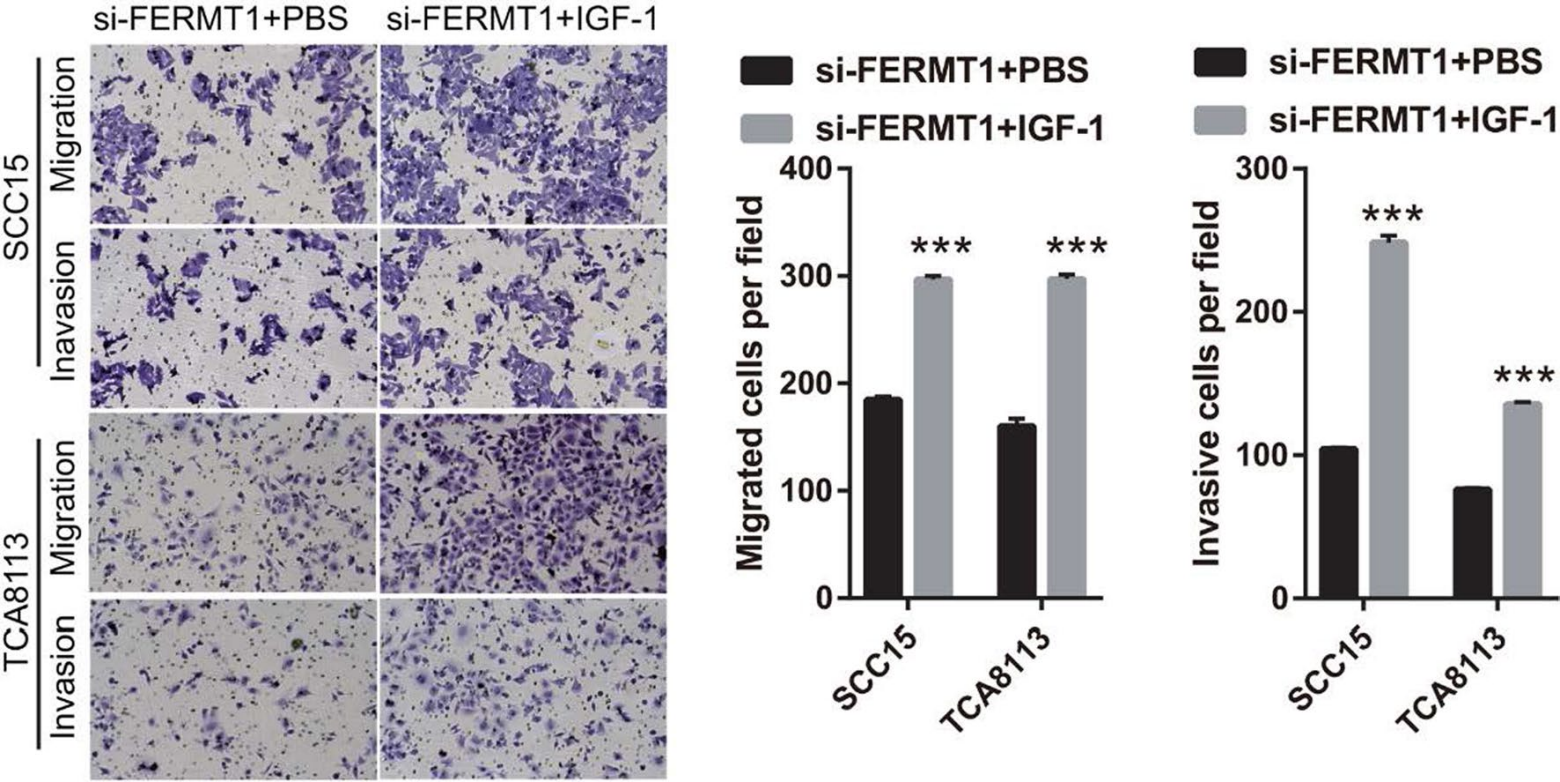
Migration and invasion were promoted after IGF-1 treatment at 24 h in si-FERMT1-transfected Tca8113 and SCC15 cells. OSCC cells were treated with 5 ng/ml recombinant human TGF-β1 protein. The migration and invasion of OSSC cells were measured by Transwell (× 200). ***P < 0.001

**Fig. 7:**
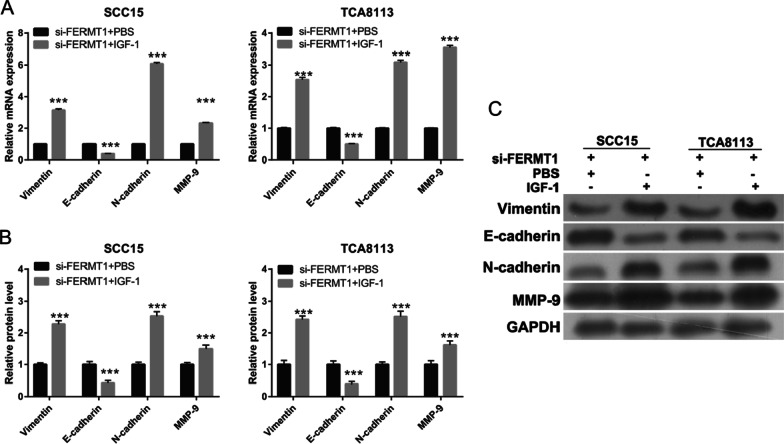
EMT was promoted after IGF-1 treatment at 24 h in si-FERMT1-transfected Tca8113 and SCC15 cells. OSCC cells were treated with 5 ng/ml recombinant human TGF-β1 protein. (**A**, **B**) The mRNA expression (**A**) and protein levels (**B**) of mesenchymal biomarkers including vimentin, N-cadherin, and MMP-9 and epithelial biomarkers including E-cadherin were measured by qRT-PCR and western blotting. (**C**) The representative image of western blot. ***P < 0.001, si-FERMT1 + IGF-1 vs si-FERMT1 + PBS

To determine whether FERMT1 contributed to OSSC migration and invasion via the PI3K/AKT signaling pathway, we activated the PI3K/AKT signaling pathway by treatment with IGF-1 (the PI3K/AKT signaling pathway activator) in si-FERMT1-transfected Tca8113 and SCC15 cells. After 48 h of treatment, the expression of PI3K and p-AKT was markedly higher in the IGF-1 treatment group than that in the PBS treatment group in si-FERMT1-transfected Tca8113 and SCC15 cells (Fig. 5). The migration and invasion of si-FERMT1-transfected Tca8113 and SCC15 cells were significantly promoted by IGF-1 treatment compared with PBS treatment (Fig. 6). Additionally, IGF-1 treatment significantly enhanced vimentin, N-cadherin, and MMP-9 mRNA expression and protein level while reduced E-cadherin mRNA expression and protein level compared with PBS treatment in si-FERMT1-transfected Tca8113 and SCC15 cells (Fig. 7).

## Discussion

The metastasis of oral cancer is one of the main causes of death [8, 9]. However, the mechanisms underlying oral cancer metastasis remain to be elucidated. In this study, we found that FERMT1 expression was elevated in TGF-β1-induced OSCC cell lines, and knockdown of FERMT1 inhibited the migration, invasion, and EMT in TGF-β1-induced OSCC cells. Notably, we revealed that FERMT1 activated the PI3K/AKT signaling pathway to promote EMT in OSCC metastasis.

FERMT1 encodes the kindlin-1 protein, which is a focal adhesion protein. A previous study found that FERMT1 expression was increased in colon cancer, which was an independent prognostic factor for poor overall survival, and FERMT1 promoted colon cancer metastasis [22, 23]. FERMT1 expression was correlated with metastasis-free survival in breast cancer, and silencing of FERMT1 inhibited lung metastasis of breast cancer [24]. FERMT1 expression was also increased in pancreatic cancer, and FERMT1 promoted pancreatic cancer metastasis [25]. These results suggest that FERMT1 expression is elevated in cancer, and that knockdown of FERMT1 expression can inhibit the migration and invasion of cancer cells. Similar to the findings of these results, we found that FERMT1 expression was elevated in TGF-β1-induced OSCC cell lines, and knockdown of FERMT1 inhibited the migration and invasion in the TGF-β1-induced OSCC cells.

EMT plays an important role in initiating metastasis. When EMT occurs, expression levels of mesenchymal biomarkers, including N-cadherin, vimentin, and MMP-9 are increased, and those of epithelial biomarkers such as E-cadherin are reduced [26]. FERMT1 mediates the β-catenin/EMT signaling pathway to promote colon cancer metastasis [27]. Additionally, Kindlin-1 is required for colorectal cancer cell migration and invasion via activation of the TGF-β/Smad3 signaling pathway and EMT [28]. Higher FERMT1 expression was found in human gastric cancer tissues and has been significantly associated with poor overall survival; FERMT1 enhanced gastric cancer metastasis and EMT by activating the NF-κB pathway [29]. These studies suggested that FERMT1 promoted cancer progression by enhancing many signaling pathways and EMT. Similar to the findings of these studies, we found that silencing of FERMT1 markedly inhibited vimentin, N-cadherin, and MMP-9 expression and promoted E-cadherin expression, suggesting that FERMT1 silencing inhibited EMT in TGF-β1-induced OSCC cells.

The PI3K/AKT pathway has been noted in OSCC and regulates cancer invasion, metastasis, and EMT [30]. In this study, we found that FERMT1 silencing inhibited PI3K and p-AKT expression, suggested that knockdown of FERMT1 silenced PI3K/AKT pathway. Additionally, activation of the PI3K/AKT signaling pathway reversed the effect of FERMT1 silencing on OSCC cell migration, invasion, and EMT. These results showed that FERMT1 regulated the migration, invasion, and EMT in TGF-β1-induced OSCC cells by activating the PI3K/AKT signaling pathway.

## Conclusion

Silencing of FERMT1 inhibits the migration, invasion, and EMT in TGF-β1-induced OSCC cells via inactivation of the PI3K/AKT signaling pathway, suggesting that FERMT1 is a novel and potential therapeutic target for anti-metastatic strategies for OSCC.

## Data Availability

All data generated or analyzed during this study are included in this published article.

## Abbreviations

FERMT1: Fermitin family member 1
OSCC: Oral squamous cell cancer
EMT: Epithelial-mesenchymal transition
TGF-β1: Transforming growth factor-β1
MMP-9: Matrix metalloproteinase 9
IGF-1: Insulin-like growth factor-1
